# Metabolic Phenotyping in Prostate Cancer Using Multi-Omics Approaches

**DOI:** 10.3390/cancers14030596

**Published:** 2022-01-25

**Authors:** Nuria Gómez-Cebrián, José Luis Poveda, Antonio Pineda-Lucena, Leonor Puchades-Carrasco

**Affiliations:** 1 Drug Discovery Unit, Instituto de Investigación Sanitaria La Fe, 46026 Valencia, Spain; nuria_gomez@iislafe.es; 2 Pharmacy Department, Hospital Universitario y Politécnico La Fe, 46026 Valencia, Spain; poveda_josand@gva.es; 3 Molecular Therapeutics Program, Centro de Investigación Médica Aplicada, 31008 Navarra, Spain

**Keywords:** prostate cancer, metabolism, multi-omics, metabolomics

## Abstract

**Simple Summary:**

Prostate cancer (PCa) is a hormone-dependent tumor characterized by a highly heterogeneous clinical outcome. This neoplastic process has become a leading cause of cancer worldwide, with over 1.4 million new cases and a total of 375,000 deaths in 2020. Despite the efforts to improve the diagnosis, risk stratification, and treatment of PCa patients, a number of challenges still need to be addressed. In this context, integration of different multi-omics datasets may represent a powerful approach for the development of novel metabolic signatures that could contribute to the clinical management of PCa patients. This review aims to provide the most relevant findings of recently published multi-omics studies with a particular focus on describing the metabolic alterations associated with PCa.

**Abstract:**

Prostate cancer (PCa), one of the most frequently diagnosed cancers among men worldwide, is characterized by a diverse biological heterogeneity. It is well known that PCa cells rewire their cellular metabolism to meet the higher demands required for survival, proliferation, and invasion. In this context, a deeper understanding of metabolic reprogramming, an emerging hallmark of cancer, could provide novel opportunities for cancer diagnosis, prognosis, and treatment. In this setting, multi-omics data integration approaches, including genomics, epigenomics, transcriptomics, proteomics, lipidomics, and metabolomics, could offer unprecedented opportunities for uncovering the molecular changes underlying metabolic rewiring in complex diseases, such as PCa. Recent studies, focused on the integrated analysis of multi-omics data derived from PCa patients, have in fact revealed new insights into specific metabolic reprogramming events and vulnerabilities that have the potential to better guide therapy and improve outcomes for patients. This review aims to provide an up-to-date summary of multi-omics studies focused on the characterization of the metabolomic phenotype of PCa, as well as an in-depth analysis of the correlation between changes identified in the multi-omics studies and the metabolic profile of PCa tumors.

## 1. Introduction

Prostate cancer (PCa) is the second most frequent cancer and represents the fifth leading cause of cancer-related death in men worldwide [1]. According to the Global Cancer Incidence, Mortality, and Prevalence (GLOBOCAN) database, new PCa cases were estimated to account for almost 1.4 million, with a total of 375,000 cancer-related deaths in 2020 [1]. Clinically, PCa is characterized by a heterogeneous behavior, ranging from indolent phenotypes to a rapid progression into an aggressive metastatic disease [2]. Early PCa diagnosis mainly relies on prostate-specific antigen (PSA) tests, although this screening method exhibits several limitations as it is prostate-specific but not cancer-specific [3], leading to overdiagnosis and overtreatment [4,5,6]. Thus, histopathological evaluation of biopsies, graded on the basis of the Gleason Score (GS) [7], is required to confirm the presence of PCa [8] and to determine the treatment strategy to follow [9]. However, prostate biopsy is an invasive procedure that might cause health complications (e.g., hematospermia, hematuria, fever, bleeding, urinary retention) [10,11]. In addition, although the grading system has been modified several times, there remains no classification scheme that allows accurately discriminating indolent from aggressive PCa stages [12]. Thus, there is a need for more precise and robust PCa biomarkers to improve diagnosis and risk stratification of patients.

In recent years, metabolic phenotyping has become a powerful approach for the identification of new molecular biomarkers and metabolic vulnerabilities that could represent novel therapeutic opportunities in oncological diseases [13,14,15,16,17,18]. Hence, several metabolomics analyses have been carried out on PCa samples (e.g., tissue, urine, serum, plasma, and seminal fluid) to characterize the specific metabolic profile associated with PCa progression and identify metabolic alterations that may potentially be used as clinical biomarkers (reviewed in [19,20,21,22]). Together, these studies have revealed a specific metabolic phenotype that could distinguish between healthy and PCa samples [23]. Healthy prostate cells accumulate high concentrations of zinc, which results in the inhibition of mitochondrial aconitase (ACO2) and consequently decreases citrate oxidation, thus disrupting the tricarboxylic acid (TCA) cycle metabolism [24]. In contrast, decreased zinc levels in PCa tumors enable the activation of ACO2 for citrate oxidation and subsequent re-establishment of the TCA cycle [23,25]. In line with this, metabolic studies have reported decreased citrate levels and increased concentrations of several TCA cycle intermediates (e.g., fumarate, malate, and succinate) in PCa tumor samples when compared with healthy prostate tissues, suggesting an increased TCA cycle metabolism [26,27,28,29]. In addition, other studies have reported lower levels of polyamines and sarcosine metabolism (e.g., spermine, spermidine, sarcosine) [29,30,31,32,33], as well as dysregulations of several amino acids (e.g., alanine, glutamate, arginine, tyrosine, phenylalanine) [26,27,28,34,35,36,37,38,39] and other metabolites involved in cellular membrane metabolism (e.g., choline, phospholipids) [26,27,40,41,42,43,44].

These metabolic alterations have been observed at different omics levels [45,46,47]. For instance, transcriptomics analyses facilitated the identification of three distinct metabolism-associated PCa clusters and the development of a six-gene metabolic signature associated with disease-free survival [47]. In addition, following a loss-of-function genetic screen, the glycolytic 6-phosphofructo-2-kinase/fructose-2,6-biphosphatase 4 (*PFKFB4*) enzyme was identified as an essential gene for PCa cell survival and evaluated as a potential therapeutic target for PCa treatment [48]. On the other hand, proteomics analyses carried out on PCa cell lines and tissue samples revealed that enzymes involved in the ketogenic metabolism pathway were overexpressed in high-grade PCa [49]. Furthermore, the characterization of the proteomics landscape of exosomes, isolated from primary prostate epithelial and PCa cell lines, identified four exosomal proteins (PDCD6IP, FASN, XPO1, and ENO1) as potential new candidate biomarkers for PCa [50]. Moreover, lipidomics, an emerging omics approach [51], has also demonstrated its potential as an alternative diagnostic tool in PCa, revealing specific associations between alterations in glycerophospholipid metabolism and fatty-acid synthesis and oxidation with PCa progression [52,53].

**Figure 1:**
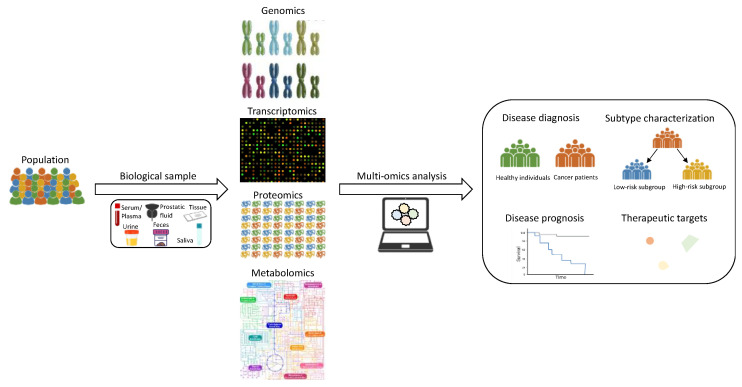
Graphical representation of different omics-based approaches and multi-omics analyses applied to the characterization of PCa-related metabolic alterations.

In summary, the information derived from different omics studies offers new avenues for better understanding the biological and molecular processes underlying metabolic changes occurring during cancer progression, as well as for developing novel molecular biomarkers to improve the clinical management of cancer patients. Moreover, a number of studies have demonstrated that the combination of multi-omics data can provide deeper insight into the metabolic changes associated with the progression of different oncological diseases than any of these omics on their own [54,55,56,57]. Thus, the integration of different omics platforms has emerged as a powerful and promising strategy for the elucidation of potential genetic and epigenetic alterations, changes in gene expression levels and signaling pathways, and other biological dysregulations that could be driving metabolic rewiring during cancer progression. Hence, this review aims to provide the most relevant findings reported in recently published multi-omics-based studies focused on the analysis of metabolic alterations associated with PCa initiation and progression (Figure 1). To that end, a literature search was conducted on PubMed, using different combinations of the following terms: “(omics OR multi-omics OR omics integration OR (metabolomics OR lipidomics) AND (metabolomics OR lipidomics OR transcriptomics OR genomics OR epigenomics OR proteomics) AND (metabol * OR metabolic profiling OR metabolic phenotype)) AND prostate cancer”. Then, titles and abstracts of the selected publications were examined to evaluate their eligibility according to their relevance on the issue of interest and to determine their inclusion in the review.

## 2. PCa Multi-Omics Studies

Between 2013 and 2021, 21 studies, focused on characterizing the specific metabolic profile associated with PCa, reported the integration of data from at least two different omics platforms. Tissue was by far the most frequently analyzed sample (15 studies), cell lines were harvested in four studies, biofluids (urine, serum) from Pca patients were collected in three of the studies, and only one of them relied on using murine models. Integration of transcriptomics and metabolomics was conducted in most of the studies, while transcriptomics, metabolomics, and lipidomics were only combined in two of them. Lastly, mass spectroscopy (MS) was preferentially chosen over nuclear magnetic resonance (NMR) as analytical platform for proteomics, metabolomics, or lipidomics analyses.

### 2.1. Benign Tissue vs. PCa Tumor

Nine of the studies discussed in this review relied on the analysis of benign prostate and PCa samples for identifying specific metabolic alterations associated with the metabolic phenotype of PCa patients (Table 1). Integration of transcriptomics and metabolomics data was the primary approach followed in these studies, and tissue samples were the biological specimens preferentially collected.

Several of these studies reported alterations in enzymes and/or metabolites involved in fatty-acid metabolism. Among them, Meller et al. observed a highly deregulated metabolism of fatty acids, sphingolipids, and polyamines in malignant tissue [58]. Altered fatty-acid and sphingolipid metabolism was associated with increased expression of acetyl-CoA carboxylase (*ACC*), ATP citrate lyase (*ACLY*), fatty-acid synthase (*FASN*), and acyl-CoA desaturase (*SCD*) as well as with elevated concentrations of several fatty acids, such as 2-hydroxybehenic acid, cerebronic acid, glycerol phosphate, and palmitic acid. Furthermore, a higher ratio of reduced (GSH) to oxidized (GSSG) glutathione and alterations in the levels of several metabolites involved in polyamine metabolism, including putrescine, spermine, and spermidine, were detected in PCa tumors. These observations were based on the metabolomics, transcriptomics, and immunohistochemistry analysis of matched malignant and nonmalignant prostatectomy samples from 106 PCa patients. These results are in agreement with previous studies reporting *FASN* to be upregulated in PCa tumors [67,68,69], and *SCD* to promote PCa proliferation [70], as its inhibition resulted in a reduction in tumor growth [71]. Other studies reported higher glutathione reductase activity in PCa, leading to higher GSH levels, which could confer higher oxidative stress resistance to these tumors [72]. A recent study also showed that mTORC1 regulated polyamine synthesis as part of an essential oncogenic metabolic reprograming in PCa [73].

Dysregulated lipid metabolism in PCa was also reported by Li et al., in a study focused on understanding the regulatory networks involved in adaptative transformation of lipid metabolism in PCa tissues [59]. Following a network-wide integrated mapping of lipid metabolism, including changes in the lipidome, transcript alterations, and post-transcriptional regulations, the authors observed a significant upregulation of de novo lipogenesis and a strengthened biosynthesis of phospholipids (PLs) via a de novo pathway in PCa lipogenesis, together with a reprogrammed composition in membrane PLs. Overall, percentages of free mono- and polyunsaturated fatty acids (MUFAs and PUFAs, respectively) were elevated, while free saturated fatty acids (SFA) were reduced. Moreover, activated PL remodeling was characterized by enhanced activities of phospholipase A2 (*PLA2s*) and reduced lysophospholipid acyltransferase (*LPLATs*), which contributed to increased MUFA-acyl residues and reduced PUFA-acyl and ether-linked chains in PCa PLs. In fact, lipogenesis upregulation has been described as a hallmark of invasive cancers and termed the “lipogenic phenotype” [74]. Furthermore, several studies have associated changes in the PL content of the cellular membrane with PCa aggressiveness [27,75,76].

The characterization of relevant master regulators contributing to the metabolic switch in PCa was also evaluated in a multi-omics study conducted by Torrano et al. [60]. In this study, the analysis of the expression levels of several metabolic coregulators in five different PCa datasets revealed that only alterations in the transcriptional coactivator PPARG coactivator 1 alpha (*PPARGC1A* or *PGC1A*), PPARG coactivator 1 beta (*PPARGC1B* or *PGC1B*), and histone deacetylase 1 (*HDAC1*) expression were present in the majority or all datasets. Among them, *PGC1A* was the only coregulator negatively associated with GS. Additional integrative metabolomics analysis demonstrated that the tumor suppressive activity of *PGC1A* was associated with a global metabolic rewiring, leading to an enhanced fatty-acid β-oxidation and TCA cycle activity. TCA cycle downregulation has also been associated with PCa progression in other multi-omics studies [77], while upregulation of TCA cycle activity has been observed when comparing PCa tumor vs. adjacent prostate tissue [62]. Notably, the results from these studies are in agreement with a previously undescribed two-step metabolic shift in the TCA cycle during PCa development and progression, which was recently identified by Latonen et al. [77]. Further in vitro and in vivo analyses performed in this study demonstrated the role of *PGC1A* in tumor progression and metastatic dissemination, with these results also being in agreement with recent findings [78]. Moreover, a recent study showed that downregulation of *PGC1A* could promote PCa aggressiveness through activation of the polyamine pathway [79].

The comparison of benign and PCa tissue samples has also revealed additional changes in energy-related metabolic pathways. Thus, in a study conducted by Lima et al., an analysis of the metabolomics and lipidomics profiles of benign and PCa tissues by NMR and MS revealed metabolic dysregulations associated with PCa development [61]. The multivariate statistical analyses revealed that the levels of 26 metabolites, including different amino acids, organic acids, and nucleotide derivatives, and 21 phospholipid species were significantly altered between both groups. Furthermore, a metabolic pathway analysis revealed 11 dysregulated metabolic pathways associated with PCa development. Dysregulations in these pathways were confirmed by strong correlations among metabolites participating in the same pathway. The main metabolic pathways associated with PCa were amino-acid metabolism, nicotinate and nicotinamide metabolism, purine metabolism, and glycerophospholipid metabolism. Notably, metabolites involved in these pathways were upregulated in PCa tissues, being in accordance with other results published in previous studies [21,22,23]. Many of these pathways provide metabolic intermediates for the TCA cycle, nucleotide synthesis, and lipid synthesis, thus contributing to the production of high levels of cellular building blocks required for rapid proliferation of cancer cells [13,80].

Shao et al. also reported accumulation and upregulation of metabolites and genes related to the TCA cycle in another multi-omics-based study [62]. Metabolomics and transcriptomics analysis of PCa tumors and matched adjacent normal tissues revealed significant accumulations of key TCA metabolic intermediates (malate, fumarate, succinate, and 2-hydroxyglutaric acid) and enrichment in genes from different anaplerotic routes, including those involved in pyruvate, glutamine catabolism, and branched-chain amino-acid (BCAA) degradation. Associations between TCA cycle and the potential anaplerotic routes were supported by increased expression of pyruvate dehydrogenase (*PDH*) complex, higher expression levels of different BCAA degradation genes, glutaminase (*GLS*) and glutamate dehydrogenase (*GLUD1* and *GLUD2*), and higher α-ketoglutarate, glutamine, and glutamate levels. Dysregulations in the TCA cycle were also identified in PCa tissues by Tessem et al. after accounting for the confounding effect of stroma [63]. In this study, integration of metabolomics and transcriptomics data revealed associations between increased succinate levels, also observed in other studies [29,81], and downregulation of succinate-CoA ligase ADP-forming subunit beta (*SUCLA2*) and succinate dehydrogenase complex subunit D (*SDHD*). Additional observations included lower citrate levels and decreased expression of *ACO1*, together with overexpression of fatty-acid synthesis genes *ACLY*, acetyl-CoA carboxylase alpha (*ACACA*), and *FASN*, suggesting an enhanced fatty-acid synthesis in these tissues. Reduced citrate concentrations and increased lipid synthesis are considered relevant metabolic features of PCa [23,82]. Furthermore, the authors observed relevant associations between reduced putrescine levels and upregulation of spermidine synthase (*SRM*), as well as lower spermine and increased spermidine/spermine *N*1-acetyltransferase 1 (*SAT1*) and spermine oxidase (*SMOX*) expression. In agreement with these results, other authors also reported a reduction in spermine and putrescine levels [34,58,74,75], as well as an overexpression of enzymes involved in the polyamine pathway [83,84,85].

In another study conducted by Kaushik et al., transcriptomics and metabolomics analyses were integrated, using a pathway-centric analytical framework that enabled the combination of the rankings of biochemical pathways enriched independently by gene expression and metabolic profiles in a single significance score [64]. Following this analysis, the hexosamine biosynthesis pathway (HBP) was found to be the most enriched pathway in treatment-naïve localized PCa, when compared to benign adjacent prostate tissues. Moreover, in silico analysis showed that the expression of glucosamine-phosphate *N*-acetyltransferase 1 (*GNPNAT1*) and UDP *N*-acetyl glucosamine pyrophosphate 1 (*UAP1*) were significantly elevated in PCa tumors. In contrast, HBP genes were significantly downregulated in castrate-resistant prostate cancer (CRPC) in comparison with localized PCa. The opposite effect of the HBP on the growth of androgen-dependent PCa and CRPC cells suggests the existence of metabolic rewiring during PCa progression. Moreover, on the basis of different in vitro and in vivo approaches, the authors concluded that downregulation of HBP in CRPC cells modulates progression via either PI3K/Akt or specific protein 1 (SP1)-regulated expression of carbohydrate response element-binding protein (ChREBP), depending on the androgen receptor variant. Previous studies have also reported several metabolic rewiring mechanisms associated with different androgen receptor variants [86]. Lastly, in this study, the authors evaluated the therapeutic efficacy of UDP-GlcNAc treatment, alone and in combination with anti-androgen therapy, for the treatment of CRPC-like tumors bearing different androgen receptor variants. Notably, in vivo UDP-GlcNAc treatment significantly reduced the proliferation in all assayed CRPC-like tumors. These findings are particularly relevant as CRPC cells containing the AR-V7 variant are essentially resistant to anti-androgen therapy. Interestingly, Ren et al. also reported increased activity of the HBP in PCa compared to adjacent prostate tissues [65]. In both studies, UDP-GlcNAc, the end product of the HBP and a key substrate for the *O*-linked *N*-acetyl-glucosamine transferase (OGT), which plays a vital role in O-GlcNAcylated modification of proteins, was found to be increased in PCa tissues [64,65]. Interestingly, posttranslational *O*-GlcNAcylation of chromatin is a significant feature of enhancers in the PCa genome [46,87]. In addition to the HBP, Ren et al. reported metabolic perturbations in other metabolic pathways, including the metabolism of cysteine and methionine and nucleotide sugars, glycerophospholipids, lysine, and sphingolipids. Moreover, nine metabolites showed potential utility as metabolic PCa biomarkers. Among them, sphingosine demonstrated high specificity and sensitivity for distinguishing PCa from benign prostatic hyperplasia (BPH), particularly in patients with low PSA levels. Other metabolomics studies have also reported alterations in the HBP and sphingolipid metabolism when analyzing the metabolic profile of PCa patients [27,28].

More recently, Lee et al. carried out a transcriptomics and metabolomics analysis of urine liquid biopsies from BPH, prostatitis, and PCa patients with a focus on the identification of PCa-specific biomarkers and the discovery of novel therapeutic targets for PCa treatment [66]. Significantly enriched pathways in PCa patients included the TCA cycle and alanine, aspartate, and glutamate metabolism. Other metabolomics studies have also reported alterations in urine levels of metabolites involved in these pathways in PCa patients [36,88,89,90]. By examining the top 25 altered metabolites and corresponding genes, the authors identified a regulatory metabolic node that influenced both pathways and was mediated by changes in glutamate oxaloacetate transaminase 1 (GOT1)- and GOT2-related metabolism. Notably, *GOT1* expression was higher in PCa patients, and glutamate, the product of GOT1, also exhibited elevated levels in these patients. Moreover, knock-down of *GOT1* in LNCaP and PC3 cells resulted in a significant decrease in cell viability, consistent with previous studies where GOT1 repression suppressed tumor growth in different tumors [91,92]. Overall, these results suggest that the metabolic alterations observed in urine liquid biopsies obtained from PCa patients could reflect the specific changes already observed in PCa cells and tumors.

**Figure 2:**
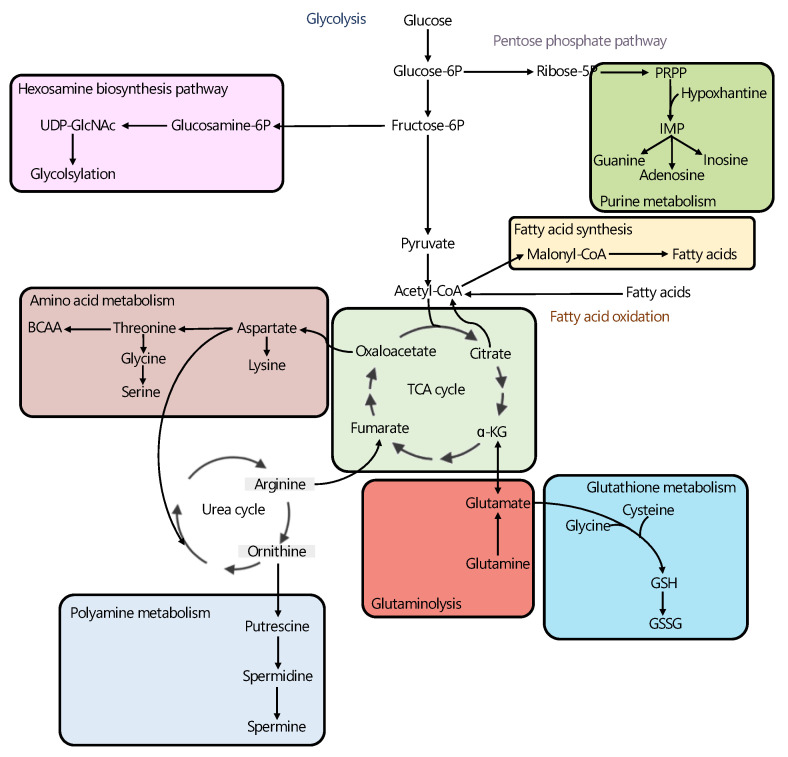
Overview of metabolic pathways most consistently reported to be altered in PCa in the multi-omics studies reviewed in this article, including: hexosamine biosynthesis pathway [64,65], purine metabolism [61], fatty acid synthesis [58,59,63], amino acid metabolism [61], TCA cycle [60,62], glutathione metabolism [58], glutaminolysis [62,66] and polyamine metabolism [58,63]. Thick lines highlight the metabolic pathways found to be upregulated in PCa tumors when compared with benign prostate tissue. References corresponding to the multi-omic studies describing alterations in each metabolic pathway are included. α-KG: alpha-ketoglutarate, Fructose-6P: fructose-6-phosphate, Glucosamine-6P: glucosamine-6-phosphate, Glucose-6P: glucose-6-phosphate, IMP: inosine monophosphate, PRPP: phosphoribosyl diphosphate, Ribose-5P: ribose-5-phosphate.

Altogether, in agreement with other studies where metabolomics was the only analytical platform used for analyzing the metabolic profile of PCa patients [39,40,41,93,94,95,96,97], the results from the multi-omics-based studies reviewed in this article suggest that the PCa-specific metabolic phenotype is characterized by alterations in the TCA cycle, polyamine synthesis, HBP, and nucleotide and lipid metabolism (Figure 2).

### 2.2. PCa Subtyping

Twelve of the multi-omics studies included in this review focused on the identification of metabolic alterations associated with specific subtypes of PCa (Table 2). Half of the studies combined transcriptomics and metabolomics analyses to characterize metabolic dysregulations in different subgroups of PCa patients, and the other half relied on the analysis of proteomics and lipidomics profiles. Tissue was the biological sample most often analyzed in these studies, whereas cell lines and biofluids were collected in only three and two studies, respectively. Different subgroups of PCa patients showed alterations in the TCA cycle and amino-acid, nucleotide, and lipid metabolism. Overall, these results correlate with metabolic changes, observed in previous studies, where metabolomics was the only analytical approach used to performed the analyses [26,27,28,75,98,99].

Several studies have revealed specific metabolic alterations in PCa patients by comparing prostate tumors of different grade. Furthermore, different systemic and local metabolic alterations have consistently been associated with PCa risk and progression [111,112,113,114,115,116]. In line with this, in a recent study by Gómez-Cebrián et al., the specific metabolomics profile of high-grade PCa patients was characterized on the basis of the alterations in metabolite levels identified in the serum and urine of PCa patients with different tumor grades [100]. A gene set enrichment analysis (GSEA) of three publicly available Pca transcriptomics datasets facilitated a targeted analysis of the metabolomics profiles, with a focus on metabolites involved in potentially altered metabolic pathways in high-grade Pca patients. Statistically significant alterations in the levels of glucose, glycine, and 1-methylnicotinamide were found in high-grade PCa patients. Interestingly, dysregulations in the levels of these metabolites could be associated with different metabolic changes previously observed in PCa patients [35,99,117,118,119]. Particularly, in other multi-omics studies based on the analysis of tissue samples, glycine levels were found to be higher in PCa tumors enriched in the TMPRSS2–ERG gene fusion set [107], and nicotinamide metabolism was elevated in PCa tissues when compared with benign tissues [61]. In addition, Kiebish et al. recently investigated the metabolic profile of presurgical serum samples of PCa patients with a focus on selecting serum metabolic biomarkers that could be valuable for predicting biochemical recurrence (BCR) [101]. In this study, the integration of proteomics, metabolomics, and lipidomics data from PCa patients facilitated the identification of four analytes (tenascin C (TNC), apolipoprotein A-IV (APOA-IV), 1-methyladenosine, and phosphatidic acid 18:0–22:0) as potential biomarkers to discriminate BCR from non-BCR patients. Of note, TNC expression levels in PCa tumor tissues and stroma have previously been reported to predict poor prognosis in PCa patients [120,121,122], and different serum studies have described apolipoproteins as a potential biomarker for PCa [123,124]. The authors evaluated the association between the levels of each individual biomarker and survival, and they found that higher levels of serum TNC, APO-AIV and 1-methyladenosine and lower concentration of phosphatidic acid increased the probability of disease progression. The predictive potential of these markers was further validated in a testing cohort of patients. Overall, the combination of the four biomolecules resulted in a model with a predictive performance for differentiating PCa patients with and without BCR characterized by an AUC of 0.78, a value that increased to 0.89 after adding the pathological T stage and the GS to the model.

Furthermore, other multi-omics studies have focused on the analysis of local metabolic changes, as reflected in the metabolic profile of PCa tissues and cell lines. In a multi-omics study conducted by Liu et al., the authors developed an approach to improve the accuracy of PCa classification and risk evaluation [102]. According to the combined analysis of genomics and metabolomics data from benign prostate samples, as well as localized and metastatic PCa samples, the authors generated classifier models that proved to be informative for Pca prognosis in additional datasets. Following this approach, they found that arginine and proline metabolism, purine metabolism, and steroid hormone biosynthesis were relevant metabolic pathways for the discrimination between localized and metastatic PCa. Next, topologically important genes and metabolites involved in these pathways were selected as promising markers for PCa prognosis. Selected genes and metabolites included cytochrome P450 family 1 subfamily A member 1 (*CYP1A1*), purine nucleoside phosphorylase (*PNP*), spermine synthase (*SMS*), proline, cholesterol, sarcosine, spermidine, and spermine. Interestingly, elevated *PNP* expression has been observed in aggressive PCa cells [125], whereas alterations in the levels of some of the topologically relevant metabolites have been associated with PCa progression and aggressiveness, including sarcosine [126,127], proline [99], and spermine [41,128]. Moreover, the classification method achieved a more accurate overall performance compared to other existing classification methods across additional datasets.

Efforts to discover dysregulated metabolic pathways in metastatic stages were also made in another multi-omics study conducted by Li et al. In this study, the authors proposed an analytical method, referred to as Subpathway-GM, aiming to identify biologically meaningful metabolic subpathways based on the combined analysis of metabolomics and transcriptomics data [103]. This method allowed the identification of disease-relevant subpathways that could go undetected on the basis of classical entire pathway identification methods. After applying this method to the analysis of a PCa dataset including data obtained from localized and metastatic tumors, 16 subpathways were identified as relevant in metastatic PCa. Among these metabolic routes, nine of them were involved in amino-acid metabolism, including glycine, serine, and threonine metabolism, tryptophan metabolism, cysteine, and methionine metabolism, and histidine metabolism. Interestingly, both tryptophan and histidine metabolism were not previously reported to be associated with metastatic PCa. Specifically, in the histidine metabolism pathway, the histamine region was accurately identified as a disease-relevant subpathway. On the basis of this information, the authors explored the effect of different histamine concentrations on PCa cell proliferation and migration. The results showed that high histamine concentrations inhibited cell migration in a dose-dependent manner, confirming that this metabolite could be associated with metastatic PCa. This finding is supplementary to other results included in previous studies where histamine altered the response to radiation in PCa tumors and significantly reduced proliferation of tumor cells compared with irradiation alone [129,130].

Other multi-omics studies have focused on the analysis of the metabolic profile associated with CRPC. Among them, the study by Latonen et al. was aimed at characterizing the distinct protein profiles of BPH, PCa, and CRPC patients [77]. To that end, the authors performed an integrated analysis of four different omics data. Following this experimental approach, it was found that gene copy number, DNA methylation, and RNA expression levels did not reliably predict proteomics changes in CRPC. These results suggested that proteomics data could be associated with alterations not detectable at the transcriptomic level. In fact, proteomics analyses revealed specific pathway alterations that were not previously reported in CRPC. Interestingly, no significant alterations were observed in the regulation of androgen receptor signaling at the mRNA or protein levels. The combined analysis by transcriptomics and proteomics identified alterations in different cell-cycle regulatory pathways, whereas changes in DNA repair pathways were only detected by proteomics. The combined analysis of the omics data also revealed a previously undescribed two-step modulation of the TCA cycle associated with metabolic changes occurring during PCa development and progression. This pathway exhibited two different metabolic shifts: a first one defined by the upregulation of most of TCA enzymes during initial PCa stages, and a second metabolic shift during PCa progression, involving the downregulation of *ACO2*, oxoglutarate dehydrogenase (*OGDH*), and succinate-CoA ligase alpha subunit (*SUCLG1*), together with elevated expression of malate dehydrogenase 2 (*MDH2*). Previous studies have already reported that PCa patients with *MDH2* overexpression have a significantly shorter period of relapse-free survival, and that stable knockdown of MDH2 PCa cell lines decreased cell proliferation and increased docetaxel sensitivity, all suggesting that MDH2 inhibition could be a viable strategy to target CRPC [131].

Additionally, other multi-omics studies have focused on characterizing metabolic dysregulations associated with specific PCa subtypes. In this context, Gao et al. integrated transcriptomics and metabolomics data to characterize the metabolic profile of two main types of PCa, adenocarcinoma (LNCaP), and small-cell neuroendocrine carcinoma (SCNC) [104]. By conducting an individual GSEA on SCNC and adenocarcinoma cell lines, a total of 62 and 112 genes, respectively, were found to be upregulated in each subgroup. Metabolomics and lipidomics analyses also revealed significant differences in 25 metabolite clusters. In particular, the LNCaP phenotype was characterized by an increased serine biosynthesis, a finding supported by elevated levels of serine, glycine, and threonine concentrations and higher expression of phosphoglycerate dehydrogenase (*PHGDH*), phosphoserine aminotransferase 1 (*PSAT1*), phosphoserine phosphatase (*PSPH*), threonine dehydrogenase (*TDH*), and glycine *C*-acetyltransferase (*GCAT*). This cell line also exhibited increased levels of citrate, isocitrate, and succinate, together with higher expression of many enzymes involved in the TCA, as well as decreased levels of fumarate, glutamate, and glutamine and lower expression of isocitrate dehydrogenase (NADP(+)) 1 (*IDH1*), *GLUD1*, and *GLUD2*, an indication of a citrate accumulation phenotype. Furthermore, an enhanced alpha-linoleic acid, arachidonic acid, linoleic acid, fatty-acid, and sphingolipid metabolism was also observed in the LNCaP group, along with a reduced fatty-acid oxidation activity, suggested by the lower levels of carnitine and some short-chain acylcarnitines and the overexpression of genes involved in biosynthesis, as well as the use of acylcarnitines and members of the acyl-coenzyme A synthetase family. On the other hand, SCNC was characterized by an enhanced glycerolipid, glycerophospholipid, and ether lipid metabolism, as well as by an elevated pyruvate metabolism, which was supported by lower levels of glucose-6-phosphate and higher lactate concentrations together with increased expression of lactate dehydrogenase isoforms (*LDHA* and *LDHB*). Although a limited number of samples were included in this pilot study, the results highlight the potential of multi-omics approaches for the identification of novel therapeutic targets in specific subgroups of PCa. Furthermore, the integrated analysis of transcriptomics and metabolomics data carried out by Joshi et al. revealed an enhanced lipid catabolism in the carnitine palmitoyl transferase I (*CPT1A*) overexpressed (OE) phenotype, which was also associated with the elevated concentration of acyl-carnitine and higher lipase activity [105]. In this study, the analysis of molecular differences between *CPT1A* gain- and loss-of-function cellular models revealed genetic and metabolomics vulnerabilities associated with the progression to neuroendocrine differentiation in PCa. Cellular models overexpressing *CPT1A* were characterized by enhanced lipid metabolism, glycine and serine metabolism, and glutathione homeostasis. In addition, the OE phenotype exhibited lower glycolysis as glucose was preferentially shunted toward de novo serine biosynthesis. This finding was correlated with the increased expression of key serine/glycine pathway genes, including *PHGDH*, *PSAT1*, and serine hydroxymethyltransferase (*SHMT2*), together with elevated levels of some metabolites involved in the folate cycle (e.g., dimethylglycine and cystathionine). Furthermore, although cells overexpressing *CPT1A* showed increased levels of mitochondrial reactive oxygen species (ROS), elevated concentrations of metabolites involved in glutathione homeostasis, including overexpression of cystathionine gamma-lyase (*CTH*) and glutathione *S*-transferase omega 2 (*GSTO2*), were also found, indicating a key role of CPT1A in supporting adaptation to stress and antioxidant defense production. Lastly, the analysis of data derived from patients, available from public databases, provided evidence that lipid catabolism driven by CPT1A was associated with more aggressive disease, suggesting that CPT1A activity could rewire metabolism to promote growth and transformation in these patients.

Other multi-omics studies have focused on characterizing the metabolic features of PCa cells undergoing epithelial–mesenchymal transition (EMT). In the study carried out by Chen et al., two subclones derived from the androgen-repressed prostate cancer cell (ARCaP) line that exhibited epithelial and mesenchymal phenotypes, ARCaP_E_ and ARCaP_M_, respectively, were used as EMT PCa models [106]. Integration of transcriptomics and metabolomics data revealed lower levels of glycolysis intermediates and decreased expression of several glucose metabolism-related genes in ARCAP_M_, indicating a downregulation of glucose metabolism. In addition, this phenotype was characterized by exhibiting higher malate levels, as well as by overexpressing *ACO2* and succinate dehydrogenase complex flavoprotein subunit A (*SDHA*) enzymes. At the same time, authors found lower succinate and citrate levels, suggesting that TCA might be fueled by glutamine and aspartate in addition to glucose in these cells. Notably, upregulation of *ACO2* has been identified as an important event in prostate carcinogenesis [23], whereas lower citrate levels have been observed in PCa when compared to non-cancer epithelium [29,132]. Furthermore, malate has been associated with Gleason progression [99] and found to be altered between different PCa stages [133]. Additionally, increased aspartate and aspartate-derived metabolite levels and upregulation of important enzymes involved in aspartate metabolism, including arginosuccinate synthase 1 (*ASS1*) and serine racemase (*SRR*), were observed in ARCaP_M_ cells, suggesting an enhanced aspartate metabolism.

A combination of metabolomics and transcriptomics data was also used by Hansen et al. to identify changes in PCa metabolism related to the TMPRSS2-ERG gene fusion [107]. In this study, PCa patients were classified in two cohorts, ERG_low_ or ERG_high_, as a function of specific enrichment of the ERG fusion gene set [134,135]. Multivariate analysis of metabolomic data revealed decreased concentrations of citrate, spermine, putrescine, and glucose, and higher levels of ethanolamine, glycine, phosphocholine, and phosphoethanolamine in ERG_high_ PCa patients included in two independent patient cohorts. Furthermore, a targeted analysis of genes involved in the metabolic pathways associated with these metabolic changes revealed an upregulation of genes involved in the polyamine pathway, together with a decrease in relevant genes in the TCA cycle and increased lipogenic phenotype. In particular, *N*(1)-acetyltransferase (*SAT1*), involved in spermine depletion, was highly expressed in ERG_high_ tumors. In addition, this group of patients also exhibited decreased expression of *ACO2* and elevated activity of the lipogenic enzymes ACACA and FASN, indicating that citrate might be preferentially derived from de novo lipid synthesis in these tumors. Several studies have also reported increased expression of *FASN* [67,68,136] and enhanced de novo fatty-acid synthesis in PCa [137] and PCa invasiveness [138]. Interestingly, in a different multi-omics study performed by Yan et al., the integration of data from three different omics platforms was used to analyze correlations between Speckle-type POZ protein (*SPOP*) mutations and changes in PCa metabolism [108]. *SPOP*, a cullin-based E3 ubiquitin ligase, has been identified as one of the most frequently mutated genes in PCa [139]. Several studies have shown that SPOP could directly bind to androgen receptor and contribute to its ubiquitination and degradation [140]. Interestingly, the authors found a strong upregulation of acyl-CoA dehydrogenase, long chain (*ACADL*), and ELOVL fatty-acid elongase 2 (*ELOVL2*) together with an increase in the levels of most fatty acids in *SPOP*-mutated patients. Relevant upregulations were also observed in the levels of two key intermediates of the TCA cycle (malate and fumarate) and fumarate hydratase (*FH*). Although *FH*, *ELOVL2*, and *ACADL* were identified as key genes in *SPOP*-mutated PCa patients in this study, their oncogenic role in PCa still needs to be proven.

Multi-omics studies have also focused on exploring specific metabolic alterations associated with PCa progression. Andersen et al. focused on identifying correlations between changes in genes and metabolites and high reactive stroma content in tumors [109], as it has been linked to worse clinical outcome and earlier BCR in Pca [141,142,143,144,145]. High reactive stroma samples were characterized by elevated levels of taurine and leucine, as well as by decreased levels of citrate, spermine, and scyllo-inositol. Interestingly, metastatic CRPC has previously been defined as leucine-dependent [146,147], and leucine deprivation has been shown to inhibit PCa growth [148]. The metabolic changes observed in high reactive stroma samples, together with the results from a gene enrichment analysis, indicated that immune processes and extracellular matrix remodeling were particularly important in these tumors. In a more recent study, Oberhuber et al. evaluated the correlation between the PCa transcriptomics and proteomics profiles with signal transducer and activator of transcription 3 (*STAT3*) expression looking for biomarkers associated with earlier BCR [110]. The integrative multi-omics analysis revealed enhanced oxidative phosphorylation (OXPHOS), TCA cycle, and ribosomal activity in the *STAT3*_low_ group of tumors. These findings were also observed in a PCa murine model, which showed enrichment of ribosomal gene sets and elevated TCA cycle and OXPHOS, as well as elevated pyruvate, fumarate, and malate levels in xenografts with loss of STAT3. The authors also observed that pyruvate dehydrogenase kinase 4 (*PDK4*) was significantly downregulated in *STAT3*_low_ samples. Expression of *PDK4* has already been reported to be significantly altered when comparing PCa patients with healthy individuals [149]. The analysis of the correlation between *PDK4* expression and BCR in primary and metastatic tumors demonstrated its ability to predict disease recurrence independently of diagnostic risk factors, such as grading, staging, and PSA levels, thus suggesting its potential as a promising independent prognostic biomarker for distinguishing between a good and bad prognostic PCa.

## 3. Future Perspectives and Conclusions

Altered cell metabolism is a well-established hallmark of cancer [150]. Metabolism is dysregulated to support the metabolic requirements of uncontrolled proliferation in cancer cells [151,152]. This rewiring of cellular metabolism leads to characteristic metabolic phenotypes that can be used for the development of effective screening methods for early cancer detection, patient selection strategies, or evaluation of treatment responses [153,154]. Altered metabolism also results in unique metabolic vulnerabilities that can be exploited to develop novel therapeutic strategies in cancer, some of which are being evaluated in preclinical models or clinical trials [17,155,156,157]. Recently, the availability and advances in the development of different analytical platforms have prompted the application of new omics approaches for the characterization of specific cancer-associated metabolic phenotypes. Particularly, metabolomics approaches have greatly contributed to metabolically characterize the profile of PCa patients and to discover specific alterations associated with this disease [22,158,159]. However, compared with other omics (e.g., genomics, transcriptomics), the metabolome coverage is limited, thus adding difficulty to the final interpretation of the results [160]. In this context, the integration of different omics datasets could represent a powerful strategy to develop more robust and consistent metabolic signatures with a clinical impact on the management of cancer patients [161].

In this review, the most relevant findings reported in multi-omics studies focused on the characterization of the metabolic phenotype associated with PCa were summarized. Overall, the most frequently reported metabolic alterations associated with PCa onset and progression include differences in the TCA cycle, polyamine synthesis, HBP, and nucleotide and lipid metabolism, and the most widely applied multi-omics approach was the combination of transcriptomics and metabolomics data. In most of the reviewed studies, the different omics datasets were separately analyzed and only combined for the final interpretation of the metabolic changes. In this scenario, the development and implementation of novel computational tools, focused on the integrated analysis of different omics datasets that enable the assessment of the interplay between the different components of a biological system, would be greatly valuable [162,163]. Furthermore, although some studies included a vast number of samples [61,63,101,107,109], a major limitation in the majority of these studies was the lack of an external cohort of PCa patients/samples for confirming the reproducibility and robustness of the results. Thus, future studies including larger sample sizes and external datasets to increase the statistical power of the analyses and validate the findings of selected metabolites, together with confirmatory experiments to evaluate the clinical significance of these metabolic findings, are required. Lastly, access to publicly accessible databases integrating all metabolic alterations reported in the literature, associated with each tumor subtype, would greatly contribute to our understanding of the metabolic heterogeneity in PCa [164,165].

## Figures and Tables

**Table 1:** Most relevant metabolic alterations reported in recent multi-omics studies focused on the characterization of the specific metabolic phenotype of PCa patients.

Study	Sample	Omics Data	Major Findings *
Meller et al. [58]	Tissue	M + T	↑ *ACC*, *ACLY*, *FASN*, *SCD*, 2-hydroxybehenic acid, cerebronic acid, glycerol phosphate, palmitic acid, GSH/GSSG, and spermidine ↓ putrescine and spermine
Li et al. [59]	Tissue	L + T	↑ *PLA2s*, free MUFA and PUFA, and *LPLATs* ↓ free SFA, PUFA-acyl, and ether-linked chains in PLs
Torrano et al. [60]	Cell lines	M + T	↓ *PGC1A,* FAO, and TCA cycle
Lima et al. [61]	Tissue	L + M	↑ amino-acid metabolism, nicotinate and nicotinamide metabolism, purine metabolism, and glycerophospholipid metabolism
Shao et al. [62]	Tissue	M + T	↑ fumarate, malate, succinate, 2-hydroxyglutaric acid, 2-ketoglutarate, glutamine, glutamate, PDH, GLS, GLUD1, GLUD2, and BCAA degradation enzymes
Tessem et al. [63]	Tissue	M + T	↑ *ACLY*, *ACACA, FASN, SAT1, SMOX, SRM*, and succinate, ↓ *ACO1*, *SDHD, SUCLA2*, putrescine, and citrate
Kaushik et al. [64]	Tissue	M + T	↑ HBP, *GNPNAT1*, *UAP1*, and UDP-GlcNAc
Ren et al. [65]	Tissue	M + T	↑ HBP, UDP-GlcNAc, and sphingosine
Lee et al. [66]	Urine	M + T	↑ *GOT1* and glutamate

*ACACA*: acetyl-CoA carboxylase alpha, *ACC*: acetyl-CoA carboxylase, *ACLY*: ATP citrate lyase, *ACO1*: aconitase, BCAA: branched-chain amino acids, FAO: fatty-acid oxidation, *FASN*: fatty-acid synthase, GLS: glutaminase, GLUD1: glutamate dehydrogenase 1, GLUD2: glutamate dehydrogenase 2, *GNPNAT1*: glucosamine-phosphate *N*-acetyltransferase 1, *GOT1*: glutamate oxaloacetate transaminase 1, GSH: reduced glutathione, GSSG: oxidized glutathione, HBP: hexosamine biosynthesis pathway, L: lipidomics, *LPLATs*: lysophospholipid acyltransferase, M: metabolomics, MUFA: mono-unsaturated fatty acids, PDH: pyruvate dehydrogenase, *PGC1A*: PPARG coactivator 1 alpha, PLs: phospholipids, *PLA2s*: phospholipase A2, PUFA: polyunsaturated fatty acids, *SAT1*: spermidine/spermine *N*1-acetyltransferase 1, *SCD*: acyl-CoA desaturase, *SDHD*: succinate dehydrogenase complex subunit D, SFA: saturated fatty acids, *SMOX*: spermine oxidase, *SRM*: spermidine synthase, *SUCLA2*: succinate-CoA ligase ADP-forming beta subunit, T: transcriptomics, TCA: tricarboxylic acid, UAP1: UDP *N*-acetyl glucosamine pyrophosphate 1. * Direction of variation, considering the benign group as reference. Up and down arrows indicate direction of the variation observed in PCa samples.

**Table 2:** Most relevant metabolic alterations reported in recent multi-omics studies focused on the characterization the metabolic phenotypes of different PCa subtypes.

Study	Sample	Omics Data	Group Comparison	Major Findings
Gómez-Cebrián et al. [100]	Urine, serum	M + T	Low- vs. high- grade PCa	High-grade: ↑ glucose, glycine, and 1-methylnicotinamide
Kiebish et al. [101]	Serum	L + M + P	non-BCR vs. BCR	BCR: ↑ TNC, APOA-IV, and 1-methyladenosine and ↓ phosphatidic acid
Liu et al. [102]	Tissue	G + M	PCa vs. metastatic	Metastatic Pca: ↑ *CYP1A1*, *PNP*, *SMS*, proline, cholesterol, sarcosine, spermidine, and spermine
Li et al. [103]	Tissue	M + T	PCa vs.metastatic	Metastatic PCa: ↓ histamine
Latonen et al. [77]	Tissue	E + G + P + T	PCa vs. CRPC	CRPC: ↓ *ACO2*, *OGDH*, *SUCLG1*, and *IDH3A*; ↑ *MDH2*
Gao et al. [104]	Cell lines	L + M + T	LNCaP vs. SCNC	LNCaP: ↑ *PHGDH*, *PSAT1*, *PSPH*, *TDH*, *GCAT*, citrate, isocitrate, and succinate; ↓ fumarate, glutamate, glutamine, *IDH1*, *GLUD1*, *GLUD2*, carnitine, and short-chain acylcarnitines SCNC: ↑ lactate and *LDH*; ↓ G6P
Joshi et al. [105]	Cell lines	M + T	*CPT1A* KD vs. *CPT1A* OE	*CPT1A* OE: ↑ *PHGDH*, *PSAT1*, *SHMT2, CTH*, *GSTO2*, dimethylglycine, cystathionine, cystathionine, and cysteine; ↓ glycolysis
Chen et al. [106]	Cell lines	M + T	ARCaP_E_ vs. ARCaP_M_	ARCaP_M_: ↑malate, *ACO2*, *SDHA*, aspartate, *ASS1*, and *SRR*; ↓ glycolysis, succinate, and citrate
Hansen et al. [107]	Tissue	L + M	*ERG*_low_ vs. *ERG*_high_	*ERG*_high_: ↑ ethanolamine, glycine, phosphocholine, phosphoethanolamine, *ACACA*, *FASN*, and *SAT1*; ↓ *ACO2*, citrate, spermine, putrescine, and glucose
Yan et al. [108]	Tissue	L + M + T	*SPOP wt* vs. *SPOP-mutant*	*SPOP-mutant*:↑ *ACADL*, *ELOVL2*, *FH*, fatty acids, fumarate, and malate
Andersen et al. [109]	Tissue	M + T	Low vs. high reactive stroma	High reactive stroma: ↑ taurine and leucine; ↓ citrate, spermine, and scyllo-inositol
Oberhuber et al. [110]	Tissue	M + P + T	*STAT3*_low_ vs. *STAT3*_high_	*STAT3*_low_: ↑ OXPHOS, TCA cycle, ribosomal activity, pyruvate, fumarate, and malate; ↓ *PDK4*

*ACACA*: acetyl-CoA carboxylase alpha, *ACADL*: acyl-CoA dehydrogenase, long chain, *ACO2*: aconitase, APO-AIV: apolipoprotein A1V, ARCaP: androgen-repressed prostate cancer cell, *ASS1*: arginosuccinate synthase 1, BCR: biochemical recurrence, *CPT1A*: carnitine palmitoyl transferase I, CRPC: castrate-resistant prostate cancer, *CTH*: cystathionine gamma-lyase, CYP1A1: cytochrome P450 family 1 subfamily A member 1, E: epigenomics, *ELOVL2*: ELOVL fatty acid elongase 2, *ERG*: ETS transcription factor ERG, *FASN*: fatty-acid synthase, *FH*: fumarate hydratase, G: genomics, *GSTO2*: glutathione *S*-transferase omega 2, *GCAT*: glycine *C*-acetyltransferase, *GLUD1*: glutamate dehydrogenase 1, *GLUD2*: glutamate dehydrogenase 2, G6P: glucose-6-phosphate, *IDH1*: isocitrate dehydrogenase (NADP(+)) 1, *IDH3A*: isocitrate dehydrogenase (NAD(+)) 3 catalytic subunit alpha, KD: knockdown, L: lipidomics, *LDH*: lactate dehydrogenase, LNCaP: lymph node carcinoma of the prostate, M: metabolomics, OE: overexpressed, *MDH2*: malate dehydrogenase 2, *OGDH*: oxoglutarate dehydrogenase, OXPHOS: oxidative phosphorylation, P: proteomics, PCa: prostate cancer, *PDK4*: pyruvate dehydrogenase kinase 4, *PHGDH*: d-3-phosphoglycerate dehydrogenase, *PNP*: purine nucleoside phosphorylase, *PSAT1*: phosphohydroxythreonine aminotransferase, *PSPH*: phosphoserine phosphatase, *SAT1*: spermidine *N*(1)-acetyltransferase, SCNC: small-cell neuroendocrine carcinoma, *SDHA*: succinate dehydrogenase complex flavoprotein subunit A, *SHMT2*: serine hydroxymethyltransferase, *SMS*: spermine synthase, *SPOP*: Speckle-type POZ protein, *SRR*: serine racemase, *STAT3*: signal transducer and activator of transcription 3, *SUCLG1*: succinate-CoA ligase alpha subunit, T: transcriptomics, TCA: tricarboxylic acid, *TDH*: threonine dehydrogenase, TNC: tenascin C.
